# Patient Navigation as an Approach to Improve the Integration of Care: The Case of NaviCare/SoinsNavi

**DOI:** 10.5334/ijic.4648

**Published:** 2019-11-15

**Authors:** Shelley Doucet, Alison Luke, Jennifer Splane, Rima Azar

**Affiliations:** 1 Jarislowsky Chair in Interprofessional Patient-Centred Care, Department of Nursing and Health Sciences, University of New Brunswick Saint John, CA; 2 Centre for Research in Integrated Care (CRIC), University of New Brunswick Saint John, CA; 3 Centre for Research in Integrated care (CRIC), New Brunswick, CA; 4 University of New Brunswick, Saint John, New Brunswick, CA; 5 Department of Psychology, Mount Allison University, CA; 6 NaviCare/SoinsNavi, Sackville, New Brunswick, CA

**Keywords:** integrated care, patient navigation, complex care, children, youth, family

## Abstract

Children and youth with complex care needs require more and varied healthcare services than the average population, as well as a high degree of coordinated care. Evidence has shown that these individuals and their families have better outcomes if they have access to integrated care. Patient navigation can serve as a novel approach to improve the integration of care for individuals with complex care needs in an increasingly fragmented system. NaviCare/SoinsNavi is an example of a navigation centre for children and youth with complex care needs, their families, and the care team. This research-based service is aimed at facilitating more convenient and integrated care using a personalized family-centred approach. NaviCare/SoinsNavi employs two patient navigators who work with clients to formulate and prioritize goals based on their unmet needs. The centre serves as a living laboratory, which provides researchers, knowledge users, and clients a *real life setting* where innovative ideas can be explored, evaluated, modified as needed throughout the research process, and moved into policy in an efficient manner. Patient navigation programs can contribute to decreasing fragmentation, improving access, and promoting integrated care across disciplines, settings, and sectors for individuals across the lifespan.

## Introduction

When caring for children and youth with complex care needs, it can be difficult and time-consuming to navigate what services and programs are available [1]. These individuals often require direct home care, hospitalizations, several emergency room visits, and/or countless appointments with specialists [2]. In addition, these children and youth and their families experience many care transitions (e.g. between providers, between settings, between stages of illness) over their lifespan and are vulnerable to gaps in care that can occur during these transitions [3]. Evidence has shown that these individuals and their families have better outcomes if they have access to integrated care [4], which refers to a person-centred system approach that is achieved through the comprehensive delivery of quality services across the life-course, designed according to the multidimensional needs of both the individual and the population, and delivered by a coordinated team of providers working across disciplines, settings, levels of care, and sectors [5].

Patient navigation programs are a novel approach to support the integration of care and transitions across the lifespan, particularly for individuals with complex care needs. The first patient navigation program was developed and implemented in 1990 in Harlem, New York, by Dr. Harold Freeman [6], who is now recognized internationally as the father of patient navigation. Patient navigation programs are increasingly being used across North America and abroad as a patient-centred approach that supports the timely movement of a client through a complex maze of fragmented services and programs across settings and sectors [6]. The patient navigator can be a professional (e.g. registered nurse, social worker), lay person (with patient navigation training), or peer (e.g. an individual who has experience living with a particular condition) [7,8]. The choice between a professional or lay navigator generally depends on the type of navigational support required, target population, and setting [7,9]. The central premise of patient navigation is to proactively guide, support, and orient patients through the healthcare system, matching patients’ unmet needs to appropriate resources to decrease fragmentation, improve access, and promote the integration of care [6,10,11].

NaviCare/SoinsNavi is a new research-based patient navigation centre in Canada that aims to facilitate more convenient and integrated care to support the physical, mental, emotional, social, cultural, and spiritual needs of children and youth up to the age of 25 and their families using a personalized family-centred model of care. Patient navigation is a relatively new concept in Canada, with NaviCare/SoinsNavi being the first navigation centre of its kind in the province of New Brunswick.

## Description of the care practice

### Development of the centre

NaviCare/SoinsNavi is a bilingual navigation centre for children and youth 25 years of age or younger with complex care needs in New Brunswick. The centre was established in January 2017 by the leadership team co-authoring this paper, thanks to a generous donation from the New Brunswick Children’s Foundation. NaviCare/SoinsNavi provides a free personalized navigational service that can be used by anyone, including youth, family members, and those involved in the care of children and youth with complex care needs (e.g. health care providers, teachers).

The services offered are based on a province-wide needs assessment, which involved over 120 interviews with youth and their families, as well as with health, social, and education stakeholders [1,12]. One of the central findings from this exploratory assessment was a need for navigational support to assist not only the families, but also members of the care team. Care providers across health, education, and social service sectors described experiencing barriers to both finding and accessing services for their clients, despite their professional expertise. When developing the centre, the NaviCare/SoinsNavi team also did multiple site visits with navigation programs across North America and conducted two environmental scans, one of services available for children and youth with complex care needs across the province of New Brunswick [1] and a second of pediatric navigation programs in Canada [13].

### Services

The centre employs two bilingual (English and French) patient navigators, one a registered nurse and the other a lay navigator, who assist youth, family members, and the care team by helping coordinate patient care; facilitating transitions in care (e.g. transitions from paediatric to adult services); connecting families with resources; helping families understand the health, education, and social services available to meet their needs; and acting as a resource for the care team. The patient navigators also help facilitate the integration of services across various levels of care, settings, and sectors, and increase community capacity in the care of children and youth with complex care needs. They identify barriers to services and programs for this population, advocate for system change, and develop evidence-based best practices.

Clients (i.e. youth, caregivers, care providers) can contact the centre’s patient navigators Monday to Friday from 9:00am-5:00pm through the centre’s toll-free number, email, or through Facebook messenger. They can reach out with a specific concern or a general need for support that can be articulated into goals that are then prioritized during an ongoing relationship with their navigator. Clients are not turned away based on their level of complexity and there is no need for a referral or diagnosis to access the centre’s services.

The process of working with the navigator begins during the first interaction, with an assessment of the client’s needs and collection of demographic information. This information is housed in the NaviCare/SoinsNavi intake platform on a secure server where data is then de-identified for research. Once unmet needs are identified by the client, with support from the navigator, goals are set to address those needs. The intervention, which involves providing personalized family-centred navigational support based on these identified goals, takes place during the course of the navigator-client relationship. The patient navigators maintain communication with the client for support and will follow-up as needed, at which time it is common for new needs to be identified. The navigators often reach out to the client’s care team to engage care providers and community stakeholders in finding a resolution to unmet needs or recognized barriers. If such system barriers and gaps in services exist and remain unresolved, they are explored and documented to be shared with decision makers. Once the client’s needs are resolved, the case is archived, although not closed, and it is understood that the client can call back at any time should new needs arise.

NaviCare/SoinsNavi has developed a policy and procedure manual to guide its operation of services and collection of client information. These documents serve as a way to systematize the collection of client data for research purposes and standardize the clinical operations of the centre, while protecting the individualized nature of care.

### Clients Served

After being in operation for two and a half years, NaviCare/SoinsNavi has served over 160 families and care providers in NB with a variety of needs, including but not limited to: finding camps, respite care, or after school care; helping to find funding; assistance in working with the care team to ensure care remains integrated; assistance in facilitating the transition from pediatric to adult care; and providing support when families are feeling overwhelmed. Families often call with several needs, or new needs may emerge as care progresses. Typically, navigators work on one to two needs per family. On average, cases are open for a period of four to seven months. The length of time cases are open is related to the complexity of the case and number of goals set with the navigator. It should be noted that much of the time that a case is open, there may be little contact with the navigator, as the navigator is spending time finding resources and services and/or trying to connect with members of the care team (as necessary). The navigators provide recommendations for services and resources located within the family’s community, across the province, and even outside of the province. Patients vary by gender, age, condition type, and region of the province. Despite this variation, the “typical” patient is male, between ages 6 to 11, with Autism Spectrum Disorder, and from one urban city in NB. Of note, the centre has also been serving families of children and youth who do not yet have a diagnosis. See Table 1 for a complete presentation of conditions, reason for calling, source of referral, and relationship to child/youth.

**Table 1:** Summary of NaviCare/SoinsNavi clients.


Conditions	**Neurodevelopmental** (e.g. **Autism Spectrum Disorder**, Intellectual Disabilities, and Cerebral Palsy); Mental Health (e.g. Attention Deficit Hyperactivity Disorder, Anxiety, and Attachment Disorder); Genetic (e.g. Rasopathies and P Ten Tumour Syndrome); Respiratory (e.g. Asthma); Gastrointestinal (e.g. Gastroparesis); Cardiac (e.g. Shome’s Complex and Heart Defect); Autoimmune (e.g. Autoimmune Dysfunction); Endocrine (e.g. PCOS); Renal (e.g. Bifold Duplex Duplicating System); Musculoskeletal (e.g. Acquired Amputation); and No Diagnosis
Reasons for calling	**Referral to Resources/Services** (e.g. **Respite Care**, Specialist Referral, Camps, Day Programs); Care Coordination; Funding; Parent/Care Provider Education; and Equipment
Referral source	**Physician**; Health Worker/Counsellor/Community Coordinator; Friend; Family; Nurse; Media; and Pamphlet
Relationship of caller to child/youth	**Mother**; Father; and Grandmother


**Note:** bold indicates most common.

### Outreach and marketing

Outreach to various stakeholders across the province has been ongoing since the launch of NaviCare/SoinsNavi in January 2017. All members of the NaviCare/SoinsNavi team (e.g. patient navigators, communications officer, co-directors, and senior research associate) have been involved in various forms of outreach and have actively built collaborative relationships across sectors. To evaluate the effectiveness of the centre’s outreach, a targeted outreach plan was developed through collaboration between the navigators and research team. Beginning with the province’s pediatricians and moving on to various other stakeholders, the centre is engaging in systematic outreach and client recruitment across the province.

In addition to building partnerships, the navigators have regular contact with the programs and services they are referring clients to, which is imperative to understanding the resources available. The programs and services identified throughout the province are compiled by the navigators into NaviCare/SoinsNavi’s resource database and uploaded to the centre’s website to support widespread ease of access. The NaviCare/SoinsNavi team has also been actively engaged in building partnerships with other patient navigation programs across Canada. These partnerships are crucial to the ongoing evolution of the centre as we can share ideas, lessons learned, and move toward building best practices for the implementation of patient navigation models of care across the lifespan for diverse populations.

Various marketing tools have been developed by NaviCare/SoinsNavi to enhance the centre’s visibility, including but not limited to a bilingual website (www.navicarenb.ca); a bilingual Facebook and Twitter page; connections articles highlighting families and care providers caring for children and youth with complex care needs; a summer camp and day program guide; a promotional video; and various print materials.

### Team

**Figure 1:**
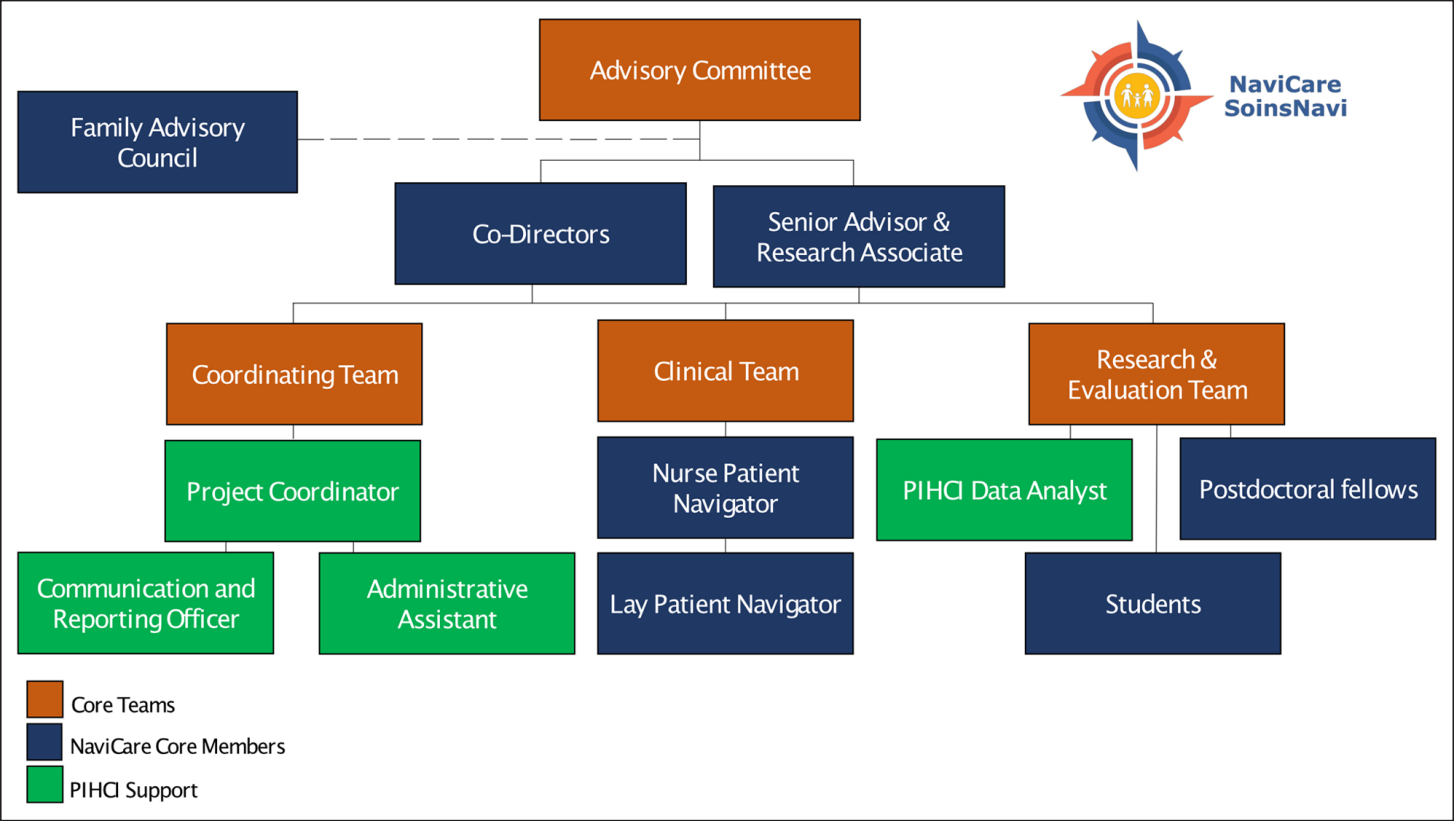
NaviCare/SoinsNavi Organizational Chart.

NaviCare/SoinsNavi began in January of 2017 with two co-directors (who are also the two co-founders) and one patient navigator and has since grown to become a diverse team of over 30 clinicians, trainees, family representatives, and researchers. A detailed overview of the centre’s organizational structure is provided in Figure 1.

The centre uses a model whereby service delivery is provided by a clinical team consisting of a professional navigator and a lay navigator. Assessment of the client is conducted by the professional navigator and then kept in her caseload or assigned to the lay navigator based on the clinical needs of the client. Both navigators engage closely with the research team to ensure a service that is both proficient in gathering relevant research data, yet also delivers individualized client-centred care.

To help ensure that NaviCare/SoinsNavi meets the needs of children and families, the centre has a *Family Advisory Council*, which includes seven volunteers. These are parents of children/youth with complex care needs or youth/adults who have experienced growing up with complex care needs. They meet monthly to advise the research team, staff, and patient navigators. The Family Advisory Council also engages in their own projects to build capacity in the community, including creating resources for families and caregivers, and developing and delivering workshops for care providers.

The centre has a productive working relationship with the *New Brunswick Network in Primary and Integrated Health Care Innovations (NB PIHCI Network)*. Through the Network, the centre has access to a data analyst, program coordinator, administrative assistant, and communication officer. The NaviCare/SoinsNavi team also has in-kind support from the University of New Brunswick’s marketing, information technology, human resource, media, and communication teams.

### Research Centre

NaviCare/SoinsNavi falls under the Centre for Research in Integrated Care (CRIC) at the University of New Brunswick. The navigation centre is operated as a living laboratory, which provides researchers, knowledge users, and patients a *real life setting* where innovative ideas can be explored, evaluated, modified as needed throughout the research process, and moved into policy in an efficient manner [14]. For example, the leadership team meets with decision makers in government to share identified gaps in services and barriers to care for this population and advocate for system change. This model serves as an ecosystem for collaboration between researchers and stakeholders to continuously develop new knowledge and innovative solutions related to integrated care.

To continue improving NaviCare/SoinsNavi, an evaluation of the short and long-term impacts of the centre is ongoing. The evaluation is informed by a linear format logic model the NaviCare/SoinsNavi team developed to support the conceptualization, organization, and planning of the NaviCare/SoinsNavi program (i.e., inputs, target populations, activities, outputs, impacts, and outcomes). All clients are required to provide consent to participate in this research centre. A mixed methods approach is being used, with data collection ranging from surveys, in-depth semi-structured interviews, client records, as well as audio/video diaries to explore the clients’ experiences over time. Where the primary purpose of our centre is to improve client outcomes and experiences (e.g. child/youth, caregiver, care provider), this has been the focus of our evaluation to date. The NaviCare/SoinsNavi research team is in the process of publishing the preliminary findings from the first two years of our centre’s implementation. In short, the findings demonstrate that families have substantial needs reflecting service gaps and barriers in care delivery across the province. Overall, families were extremely satisfied with the centre. Emerging themes include the feeling of relief to find someone who would listen, reduced feelings of stress, improved care coordination, and increased knowledge of programs and services.

Moving forward, our research team has hired a part-time data analyst who will assist us with developing metrics to evaluate and monitor the impact of NaviCare/SoinsNavi. For example, our evaluation will include measuring a number of short, medium, and long-term outcomes, such as increased knowledge of resources and services; increased ability to participate in activities at home, school, and in community; reduced stress for caregivers; and improved quality of life. Metrics are needed to evaluate whether patient navigation can improve the quality of care, user experiences, health outcomes, and cost-effectiveness of care for children and youth with complex care needs. The key metrics will include those that are most likely to be impacted by patient navigation and will take into consideration the cultural, educational, familial, financial, and other socio-demographic considerations of the population in which the patient navigation intervention is being studied. The metrics will also be chosen to support a policy-relevant research agenda to evaluate the efficacy of patient navigation and care integration. Moving forward with our evaluation, we will also track the duration of time that cases are open and break it down into number of touches with clients and minutes spent on calls and emails.

### Teaching Centre

Housed at the University of New Brunswick, NaviCare/SoinsNavi is also a teaching centre. For example, clinical trainees in nursing and medicine are engaged in shadowing our outreach operations; nursing students, masters in business administration interns, and undergraduate interns/summer students across disciplines are engaged in supporting our organizational operations; and research trainees at the undergraduate, graduate, and postdoctoral levels are engaged in evaluating the centre. The rich research and practice environment positively transforms the educational experience of the involved trainees, as they have opportunities to not only learn from the NaviCare/SoinsNavi team, but also their peers.

## Discussion

Children and youth with complex care needs require more and varied healthcare services than the average population, as well as a high degree of coordinated care. It is recognized internationally that there is a need for integrated service delivery models for this population that are community-based with integrated linkages to secondary care and tertiary care, as well as relevant sectors outside of the healthcare system [15]. Patient navigation can serve as a novel approach to improve the integration of care for children and youth with complex care needs and their families in an increasingly fragmented system.

While there are many definitions of integrated care, implicit in nearly every definition is the notion that integrated care should be centred on the needs of individuals, their families, and communities [15]. Our team developed NaviCare/SoinsNavi based on the need for navigational support, as identified by children and youth with complex care needs, their families, and stakeholders involved in their care across communities in New Brunswick. The intake process with NaviCare/SoinsNavi’s patient navigator allows for the client to articulate their needs based on the gaps and barriers faced within the system. Once their goals have been identified, the patient navigator works collaboratively with members of the care team towards meeting these goals and improving health outcomes for the client. The centre’s Family Advisory Council ensures that the centre is continuously meeting the unique needs of children and youth, their families, and the care team by sharing their lived experience and expertise. Where NaviCare/SoinsNavi was established as a living lab, this means the patient navigators provide support in a way that incorporates current research findings from the ongoing evaluation of the centre; thus continuously adapting NaviCare/SoinsNavi’s services to address any new needs or evidence over time.

Patient navigation can support the integration of care at the micro, macro, and meso-levels. Patient navigators can support integration at the *micro-level* through working with individuals to achieve a shared plan of care across providers [15]. At the *meso-level*, patient navigators can support a particular care group or population with the same condition, such as through offering capacity building with care providers who regularly care for these populations. As an example, Navicare/SoinsNavi’s Family Advisory Council is actively involved in developing capacity in the community through the development of resources and workshops for caregivers in partnership with the centre’s patient navigators. Integration at the *macro-level* can involve improving the integration of care for an entire population through the identification of needs and tailoring of services according to these needs [15]. For example, the patient navigator can work with decision makers to advocate for system change based on identified needs and gaps in services.

One of the concerns identified through the implementation of patient navigation programs is that it adds an additional role and another layer of complexity in a system that is already plagued with confusion. To address this concern, it is recommended that patient navigators have a clear scope of practice that distinguishes the roles and responsibilities of the navigator from other members of the care team [6]. It is also recommended that navigators be integrated into the care team to promote optimal benefit for the client [6]. NaviCare/SoinsNavi’s patient navigators are continually working with members of the care team to improve coordination and communication between team members and caregivers. The navigators serve as a single point of contact for children and youth, caregivers, and care providers to mitigate system disparities and ensure the integration of care.

## Conclusion

Patient navigation is a novel approach to health service delivery that can contribute to improving the integration of care across disciplines, settings, levels of care, and sectors. NaviCare/SoinsNavi is a case example of how a research-based patient navigation centre can facilitate more convenient and integrated care to support the physical, mental, emotional, social, cultural, and spiritual needs of children and youth and their families using a personalized family-centred model of care. NaviCare/SoinsNavi is also an example of how patient navigators are in a unique position as point-persons in circles of care to both identify system gaps that are detrimental to the ongoing care of this population and to forge relationships with decision-makers to advocate for system change. Although future research is still needed to inform the impact of patient navigation programs on achieving more coordinated and integrated care, the case of NaviCare/SoinsNavi demonstrates the important role of patient navigation to address fragmented care for individuals with complex care needs.
